# Influence of Grain Size on Dielectric Behavior in Lead-Free 0.5 Ba(Zr_0.2_Ti_0.8_)O_3_–0.5 (Ba_0.7_Ca_0.3_)TiO_3_ Ceramics

**DOI:** 10.3390/nano13222934

**Published:** 2023-11-12

**Authors:** Vladimir Lucian Ene, Valentin Razvan Lupu, Claudiu Vasile Condor, Roxana Elena Patru, Luminita Mirela Hrib, Luminita Amarande, Adrian Ionut Nicoara, Lucian Pintilie, Adelina-Carmen Ianculescu

**Affiliations:** 1 Department of Science and Engineering of Oxide Materials and Nanomaterials, Faculty of Chemical Engineering and Biotechnologies, National University of Science and Technology Politehnica Bucharest, 1–7 Gheorghe Polizu Street, 011061 Bucharest, Romania; vladimir.ene@upb.ro (V.L.E.); valentin.lupu2702@stud.chimie.upb.ro (V.R.L.); claudiu.condor@stud.chimie.upb.ro (C.V.C.); adrian.nicoara@upb.ro (A.I.N.); 2 National Research Center for Micro and Nanomaterials, National University of Science and Technology Politehnica Bucharest, 060042 Bucharest, Romania; 3 National Institute of Materials Physics, Atomistilor 405A, 077125 Magurele, Romania; roxana.patru@infim.ro (R.E.P.); luminita.hrib@infim.ro (L.M.H.); amarande@infim.ro (L.A.); pintilie@infim.ro (L.P.)

**Keywords:** BCT-BZT ceramics, grain size, spark-plasma sintering, conventional sintering, sol-gel

## Abstract

Fine-tuning of grain sizes can significantly influence the interaction between different dielectric phenomena, allowing the development of materials with tailored dielectric resistivity. By virtue of various synthesis mechanisms, a pathway to manipulate grain sizes and, consequently, tune the material’s dielectric response is revealed. Understanding these intricate relationships between granulation and dielectric properties can pave the way for designing and optimizing materials for specific applications where tailored dielectric responses are sought. The experimental part involved the fabrication of dense BCT-BZT ceramics with different grain sizes by varying the synthesis (conventional solid-state reaction route and sol-gel) and consolidation methods. Both consolidation methods produced well-crystallized specimens, with Ba_0.85_Ca_0.15_O_3_Ti_0.9_Zr_0.1_ (BCTZ) perovskite as the major phase. Conventional sintering resulted in microstructured and submicron-structured BCT-BZT ceramics, with average grain sizes of 2.35 μm for the solid-state sample and 0.91 μm for the sol-gel synthesized ceramic. However, spark plasma sintering produced a nanocrystalline specimen with an average grain size of 67.5 nm. As the grain size decreases, there is a noticeable decrease in the maximum permittivity, a significant reduction in dielectric losses, and a shifting of the Curie temperature towards lower values.

## 1. Introduction

Due to their flexibility in accommodating a large number of elements of the periodic system, which determines their versatile, functional properties, ferroelectric perovskites are among the most investigated categories of electroceramics. Nowadays, a particular approach that is stirring increasing interest aims to identify and develop eco-friendly perovskite materials in order to replace the toxic lead-based Pb(Zr,Ti)O_3_ (PZT) ceramics in electronic devices. BaTiO_3_-based systems represent one of the most studied classes of ferroelectric perovskites because of their unique physical and chemical properties, such as high dielectric constant, piezo and pyroelectric effect, PTCR (positive temperature coefficient of resistivity) behavior, high-voltage tunability, high endurance under DC field stress, switching phenomena, high chemical inertness, and non-toxicity, which make them useful for a large range of applications as multilayer ceramic capacitors—MLCC and embedded capacitors in printed circuit boards, energy harvester sensors and actuators, pyroelectric IR sensors, thermistors, non-volatile memories, etc. [1,2]. Site-occupancy engineering, as well as compositional design, is commonly used to obtain A-, B- or simultaneously A- and B-site homovalently substituted BaTiO_3_-based solid solutions in order to tune their physical properties and to control both the ferroelectric Curie temperature and the type of the ferroelectric-paraelectric phase transition, in such way to make these materials also suitable for energy storage applications [3].

A typical exponent among the lead-free ferroelectric perovskites is represented by the quaternary (1 − x)Ba_0.7_Ca_0.3_TiO_3_–xBaTi_0.8_Zr_0.2_O_3_ (BCT–BTZ) solid solution. Of particular interest is the 0.5Ba_0.7_Ca_0.3_TiO_3_–0.5BaTi_0.8_Zr_0.2_O_3_ (BCTZ) composition, which is placed on a morphotropic phase boundary (MPB), similar to that found in the PbZrO_3_–PbTiO_3_ (PZT) system [4], where, according to some earlier phase diagrams [5,6], the rhombohedral and tetragonal polymorphs coexist. The recently revised phase diagram of the BCT–BTZ system shows that the morphotropic BCTZ composition should consist of a mixture of orthorhombic and tetragonal modifications at room temperature [7,8]. A large number of works were dedicated to the preparation of BCTZ ceramics by the well-known conventional solid-state reaction method, reporting data regarding the related functional properties [9,10,11,12,13,14,15,16]. The values of the average grain size, permittivity maximum, and Curie temperature are spread over a large range due to the different reagents, processing factors, and especially sintering strategies used. Commonly, the classical solid-state route leads to coarser-grained ceramics with a grain size of 15–50 μm. Considering the first-order nature of the ferroelectric-paraelectric phase transition and the associated sharp permittivity maxima, it is obvious that the thermal stability of the dielectric response is strongly affected. Thus, at room temperature, the relative permittivity value drops to about a quarter of the permittivity maximum. In order to overcome this drawback, alternative wet-chemical synthesis methods, such as sol-precipitation [17], sol-gel [18,19], hydrothermal [20], microwave-assisted sol-gel hydrothermal (MSGH) [21], and molten salts routes [22], were proposed for preparing starting nanocrystalline powders of high purity. It was found that, even if consolidation by conventional sintering led to obtaining controlled and more uniform microstructures, it did not allow the granular size to drop in the submicron range. For this reason, innovative consolidation techniques, such as microwave sintering [23], plasma-assisted sintering [24,25], hybrid sintering [26], two-step sintering [27], and spark plasma sintering [27,28] were also used, to further decrease the grain size in BCTZ ceramics. Even so, only Hao et al. [27] reported an average grain size value below 1 μm (<*GS*> = of 0.4 mm) for their BCTZ specimen obtained by spark plasma sintering (SPS) at 1120 °C for 5 min. Due to the high densification involving a low porosity, irrespective of the synthesis/sintering strategy adopted, the BCTZ ceramics exhibit low values of dielectric losses (below 5%). Notably, the microstructure’s influence on the dielectric behavior was studied especially for the conventionally sintered BCTZ ceramics when grain size decreased from tens to a few microns [18,20,22].

The influence of grain size down-scaling towards the submicron and nanometer range on electrical properties was intensively studied mostly in undoped BaTiO_3_ bulk ceramics [29,30,31,32,33] and in some of the homovalent solid solutions such as (Ba,Sr)TiO_3_ [34,35,36] and Ba(Ti,Zr)O_3_ [37]. It was shown that specific features such as reduced and frozen polarization, flattening of the permittivity-temperature (*ε*(T)) maximum, reduction of both Curie temperature and dielectric constant to ~1000, and non-hysteretic permittivity-field (*ε*(E)) dependence and high tunability, all originating from complex stress-induced structural distortions associated with the dramatic drop of grain size, fulfill the requirements imposed for microwave devices [28,29,30,31,32,33,34,35,36]. A systematic study regarding the influence of the structuring level on the dielectric behavior of the BCTZ ceramics, when the grains cover a wide range of sizes, from tens of microns to tens of nanometers, has not yet been reported. Hence, the objective of this work paper is to shed light on how alternative synthesis routes and sintering techniques involving structuring at different scales impact the properties of lead-free ceramics. This involves examining the structure, microstructure, and dielectric properties of 0.5 Ba(Zr_0.2_Ti_0.8_)O_3_–0.5(Ba_0.7_Ca_0.3_)TiO_3_ ceramics prepared by both the solid-state reaction method and the sol-gel route, and consolidated through conventional and spark plasma sintering techniques.

## 2. Materials and Methods

### 2.1. Sample Preparation by Solid-State Reaction Route

**Figure 1:**
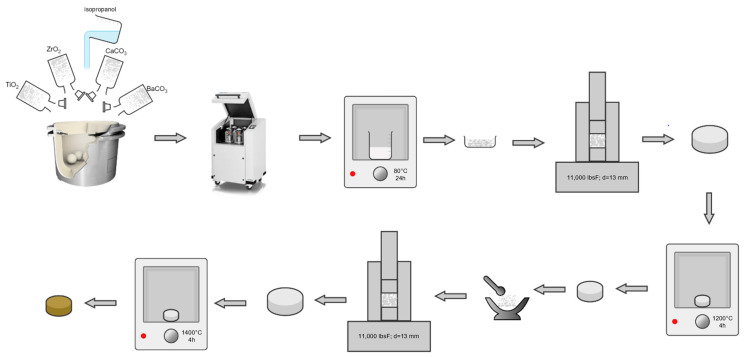
Solid-state route schematic for obtaining 0.5BCT-0.5BZT SS-CS ceramic.

For the synthesis of the 0.5 Ba(Zr_0.2_Ti_0.8_)O_3_–0.5 (Ba_0.7_Ca_0.3_)TiO_3_ system using the solid-state method, barium carbonate (BaCO_3_, 99.9% purity), zirconium dioxide (ZrO_2_, 99.9% purity), calcium carbonate (CaCO_3_, 99.9% purity) and titanium dioxide (TiO_2_, rutile, 99.9% purity) were used as the starting materials, and processed as represented in Figure 1. Stoichiometric weights of all the powders for 100 g ceramic were weighed, mixed, and ball milled with 100 mL 2-propanol (C₃H₈O, 99.99% purity) for 12 h at 400 rpm, using 10 mm agate balls as the grinding media. All chemical reagents were commercial, from Merck, former Sigma Aldrich (Darmstadt, Germany).

After homogenization in the ball mill, the suspensions were oven-dried for 24 h at 80 °C, and the balls were subsequently separated by sieving on a 1 mm mesh sieve. From the homogenous powder, 1.5 g masses were extracted, and each was mixed in an agate mortar with 5 drops of 5 wt.% polyvinyl alcohol (PVA) aqueous solution. The mixtures were then uniaxially pressed in a hydraulic press using a 13 mm diameter die and a pressure of 368 MPa. The resulting green pellets were pre-sintered in static air at 1200 °C for 4 h (with a heating rate of 5 °C/min). Pre-sintering occurs more efficiently in a green body than in powder, thus stimulating the initiation of solid phase reactions and the formation of the perovskite skeleton. The pre-sintered bodies were milled and pressed identically to the precursor powders and sintered in static air at 1400 °C with a 4 h plateau (at a heating rate of 5 °C/min). The obtained ceramic from solid-state synthesis followed by conventional sintering is further referred to as SS-CS.

### 2.2. Sample Preparation by Sol-Gel Reaction Route

**Figure 2:**
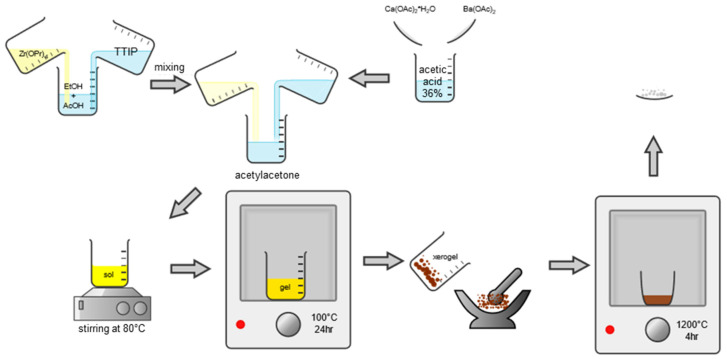
Sol-gel synthesis route schematic for obtaining 0.5BCT-0.5BZT SG powder.

**Figure 3:**
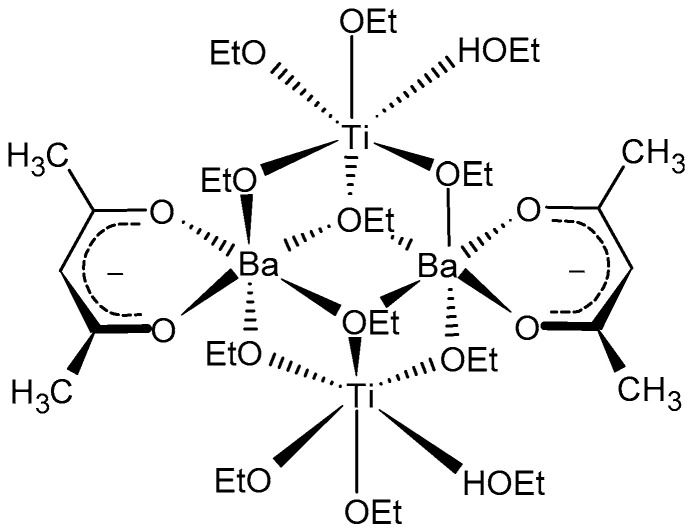
The complex combination causing stabilization, Ba centers can be substituted by Ca, analogous to Ti and Zr.

The 0.5 Ba(Zr_0.2_Ti_0.8_)O_3_–0.5 (Ba_0.7_Ca_0.3_)TiO_3_ powders were prepared using the sol-gel method with barium acetate ((CH_3_COO)_2_Ba, 99.9% purity), calcium acetate ((CH_3_COO)_2_Ca·H_2_O, 99.99% purity), zirconium (IV) propoxide solution 70 wt.% in 1-propanol (Zr(OCH_2_CH_2_CH_3_)_4_, further referred as Zr(OPr)_4_), and titanium tetraisopropoxide (Ti(OCH(CH_3_)_2_)_4_, 99.999% purity, further referred as TTIP) as starting materials, and processed as represented in Figure 2. Glacial acetic acid (CH_3_COOH, 99.7%) and 2-propanol were used as solvents, while acetyl-acetone (C_5_H_8_O_2_, 99.99% purity) was used as a stabilizing agent. Stabilization with acetyl-acetone was carried out because previous experiments had shown that after calcination, titanium-rich phases such as BT2, B2T9, and other polytitanates could be distinguished by X-ray diffraction along with free barium and calcium oxides. Acetyl-acetone remedies this problem by forming a tetranuclear complex combination with barium, calcium, titanium, and zirconium (Figure 3) [38]. All chemical reagents were commercial, from Merck, former Sigma Aldrich (Darmstadt, Germany).

Hence, in order to obtain 10 g of oxide powder, the appropriate amounts of barium and calcium acetates were placed in a beaker together with 45 mL of 36 wt.% acetic acid, ensuring complete dissolution by magnetic stirring. In another beaker, the appropriate volumes of TTIP and Zr(OPr)_4_ were placed together with 17.5 mL of 2-propanol and 8.5 mL of glacial acetic acid, then homogenized by magnetic stirring. After obtaining the two clear solutions, the alkoxide mixture was gradually added dropwise over the acetate mixture under continuous stirring. After mixing, a pale-yellow transparent solution was obtained, to which 5 mL of acetyl-acetone was added. An immediate intensification of the color was observed, the solution becoming deep yellow. The resulting sol was afterward heated to 80 °C under continuous stirring until gelation, which occurs abruptly when the optimum temperature is reached. The gel formed is oven-dried at 80 °C to obtain a dark brown xerogel, which was ground and calcined at 900 °C with the formation of the final oxide powder (SG).

Next, 2 types of consolidation methods were employed: conventional sintering (CS) and spark plasma sintering (SPS).

For the conventional sintering, batches of 1.5 g from the resulting powder were milled and shaped by uniaxial pressing at 368 MPa into pellets with a diameter of 13 mm after adding 5 drops of 5 wt.% PVA aqueous solution. The green bodies were sintered in a muffle furnace, in static air at 1400 °C for 4 h plateau, using a heating rate of 5 °C/min and slowly cooled afterward (with the normal cooling rate of the furnace) at room temperature to obtain dense BCT-BZT ceramic samples. The obtained ceramic from sol-gel synthesis followed by classical consolidation (sintering) is further referred to as SG-CS.

For SPS, batches of 1 g from the resulting powder were introduced into a 9 mm graphite mold and pressed with a force of 3.9 kN (an equivalent pressure of 61 MPa). Heating was initiated at a controlled rate of 100 °C/min until a temperature of 1050 °C was reached, maintained at this temperature for 4 min; then cooling was performed gradually over 10 min. This gradual increase in temperature was critical to prevent any sudden thermal shocks that could potentially compromise the structural integrity of the material, while the controlled cooling helped minimize any residual stresses and prevent any sudden temperature changes that could lead to cracking or other defects in the final product. The obtained ceramic from the sol-gel synthesis followed by unconventional sintering is further referred to as SG-SPS.

### 2.3. Samples Characterization

X-ray diffraction (XRD) measurements were performed at room temperature using an Empyrean diffractometer from Malvern PANalytical (Bruno, The Netherlands), operating in a Bragg–Brentano configuration with Ni-filtered Cu-Kα (λ = 1.5406 Å) radiation. The patterns were recorded in the order 10° < 2θ < 80° with a counting time per step of 255 s and a step size of 0.02°. Phase identification was performed using HighScore Plus 3.0e software connected to the ICDD PDF-4+ 2023 database. Lattice parameters were refined using the Rietveld method. The microstructure of all BCT-BZT ceramics was observed by high-resolution scanning electron microscopy, using a QUANTA INSPECT F50 microscope (FEI, Hillsboro, OR, USA) equipped with field electron emission gun (FEG) and an energy dispersive spectroscopy (EDS) detector, at an acceleration voltage of 30 KV and a point-to-point resolution of 1.2 nm. FE-SEM investigations were carried out in SE (secondary electron) mode on polished and subsequently thermally etched cross-sections. The average size of the ceramic grains was determined as the mean intercept length considering measurements on ~200 grains from images captured at similar magnifications from diverse microscopic fields through the ImageJ software. The relative density values of the sintered ceramic pellets were calculated as the ratio between the apparent density measured by Archimedes’ principle and the crystallographic (theoretical) density.

RAMAN spectroscopy was performed to achieve a more precise identification of polymorphic variants within the analyzed samples using LabRAm HR Evolution (Horiba, Lyon, France) equipment. Raman spectra were recorded using the 514 nm line of an argon ion laser by focusing a 125 mW beam of a few micrometers spot size on the samples under investigation. Data were collected and analyzed for the non-polarized radiation scattered at 90°, with an acquisition time of 10 s during 5 runs recorded for each sample.

The disc-shaped ceramics were polished to get uniform and smooth surfaces in a parallel plate configuration, essential for electrode coating. For the electric measurements, silver paste was applied onto both the top and bottom disc surfaces, and measurements were taken in a vacuum to prevent any unwanted atmospheric effects. The impedance and phase shifts were recorded using an advanced computer-controlled Impedance Analyzer (HIOKI IM3570, Nagano, Japan) across an extensive temperature range between 50 K and 470 K, maintaining a steady cooling rate of 1 K/min and an accuracy better than 0.03 K, in a frequency window set between 102 and 106 Hz under an alternating current (a.c.) of 1V.

## 3. Results and Discussion

### 3.1. Phase Purity, Structural and Microstructural Characteristics

**Figure 4:**
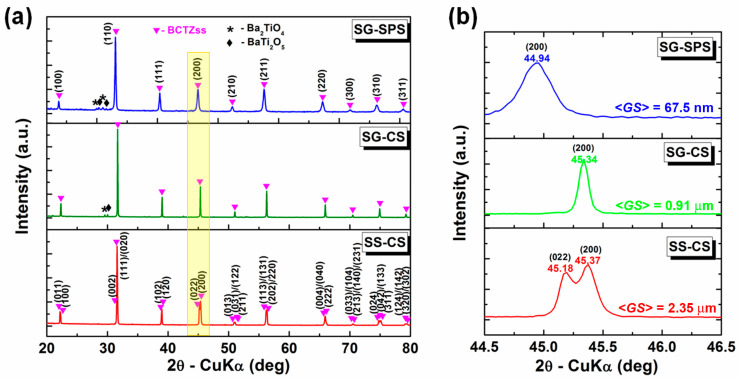
(**a**) X-ray diffraction patterns recorded at room temperature for the BCTZ ceramics obtained by alternative synthesis/sintering techniques and (**b**) detail (yellow rectangle of (**a**)) of 2θ region corresponding to the (200) reflection.

Figure 4 shows the X-ray diffraction patterns obtained for the sintered ceramic discs, which reveal that both consolidation methods produce well-crystallized specimens that tend to single-phase composition. Apart from the major Ba_0.85_Ca_0.15_O_3_Ti_0.9_Zr_0.1_ perovskite phase, only small amounts of monoclinic Ba_2_TiO_4_ (ICDD no. 04-006-8930) and orthorhombic BaTi_2_O_5_ (ICDD no. 04-017-1818) were identified at the detection limit of the diffractometer as residual phase in SG-CS and SG-SPS ceramics, frequently reported in the literature for the sol-gel synthesis [36].

The XRD maxima of the SG-SPS sample present bell-shaped profiles and reduced intensity, characteristic of a shorter-range crystalline order induced by the nanostructure, compared to the Lorentzian maxima specific to the long-range order identified in the other 2 coarser SS-CS and SG-CS samples. The detail of Figure 4b, depicting the yellow rectangle of Figure 4a corresponding to the 2θ region of 44.5–46.5° indicates that the grain size decrease induces the shift of the main reflections toward lower values of the diffraction angle, as well as a structural change from an orthorhombic unit cell (ICDD no. 01-086-8337), specific to the coarser SS-CS sample obtained by the conventional solid-state reaction method, toward a “pseudo-cubic” structure for the finer-grained SG-CS and SG-SPS ceramics. This modification is pointed out by the merger of the neighboring (022) and (200) peaks specific to a lower symmetry phase into a single (200) maximum specific to a cubic structure.

The unit cell parameters were extracted using Rietveld refinement. The structural data, as well as the values of the Rietveld fitting parameters R expected (*R*exp), R profile (*R*p), weighted R profile (*R*wp), and goodness of fit (χ^2^), are presented in Table 1. An increase in the unit cell volume of the major orthorhombic polymorph was found with decreasing grain size. This trend of the unit cell volume expansion was also observed in the (Ba_0.6_Sr_0.4_)TiO_3_ ceramics when the grain size dropped from the submicron toward the nanometer range [36]. The prevalence at room temperature of the orthorhombic form in the conventionally sintered, coarser samples is in good agreement with the recently revised phase diagram of the BCT-BTZ system, which shows an intermediate compositional range corresponding to the orthorhombic polymorph, placed between the ranges associated with the rhombohedral and tetragonal phase, respectively [7,8]. It is worth mentioning that, in the case of the nanocrystalline SG-SPS sample, the lowest values of the fitting parameters provided by the Rietveld analysis were obtained by considering that the so-called “pseudo-cubic” structure of the major perovskite BCT-BTZ phase actually represents a mixture of orthorhombic (ICDD no. 01-086-8337), tetragonal (ICDD no. 00-072-0077), and cubic (ICDD no. 00-070-0583) modifications (Table 1). The significant proportion (27.9%) of the cubic phase seems to indicate that the Curie temperature of this sample could be shifted below the room temperature, which means that it is found in the proximity of the paraelectric state. In order to have a more accurate picture of the structure, especially for both the submicron-structured (SG-CS) and nanocrystalline (SG-SPS) samples, further Raman spectroscopy analyses at room temperature and dielectric measurements versus temperature are required.

**Figure 5:**
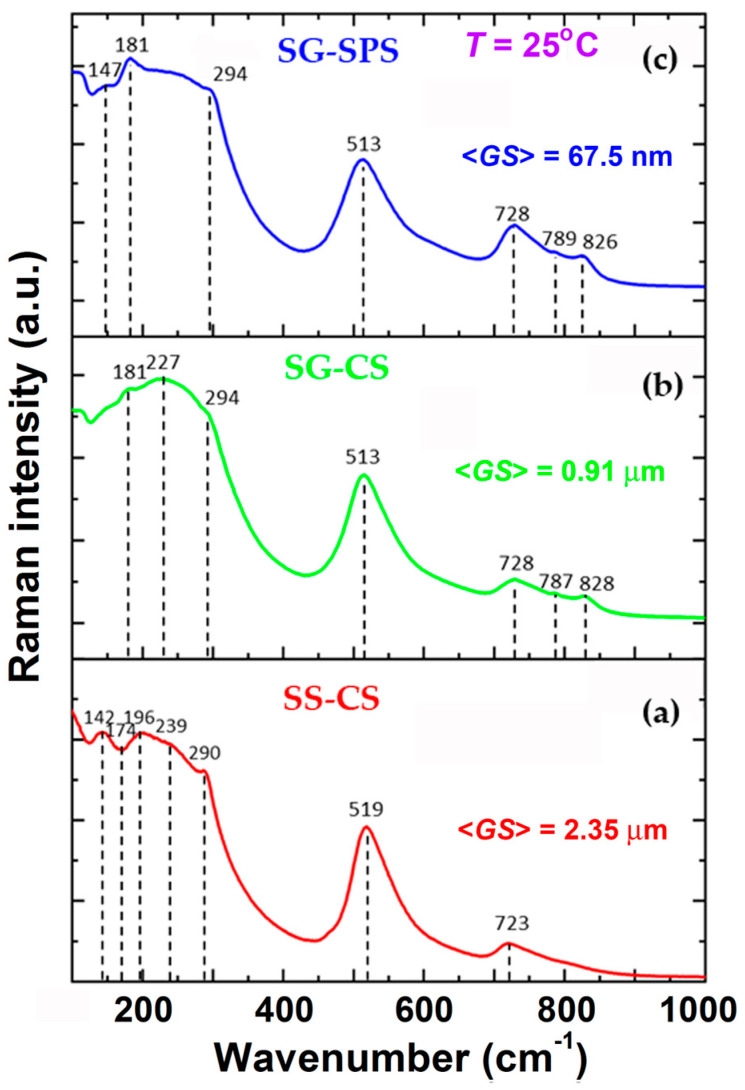
Raman spectra at room temperature for the BCTZ ceramics prepared by alternative synthesis/sintering techniques: (**a**) SS-CS, (**b**) SG-CS, and (**c**) SG-SPS.

Since X-ray diffraction averages the structure over ~10,000 elementary cells, not being able to detect local distortions present in the samples efficiently, it was necessary to perform Raman spectroscopy analysis at room temperature, sensitive to cation ordering at short distances, in order to identify polymorphic variants in the analyzed samples more accurately. In the case of the SS-CS ceramic sample, obtained by the solid-state reaction method (Figure 5a), the presence in the Raman spectrum of both the “silent” [*E*(TO) + *E*(LO) + *B*_1_] mode at 290 cm^−1^ and the interference dip at 174 cm^−1^ due to the interaction of the *A*_1_ modes, represents the signature of the tetragonal phase [39]. Moreover, the positive [*A*_1_(TO) + *E*(LO)] phonon mode at the frequency of 196 cm^−1^, visible as a shoulder in the left-side of the [*A*_1_(TO)] mode located at 239 cm^−1^ and associated with a certain flattening of the last one relative to the tetragonal phase, is attributed by some authors to the orthorhombic distortion [40,41]. These characteristics lead to the conclusion that for SS-CS, there is an overlap of phases with orthorhombic and tetragonal symmetry of the unit cell. The appearance of co-modes at wave numbers 519 cm^−1^ [*E*(TO) + *A*_1_(TO)] and 723 cm^−1^ [*A*_1_(LO), *E*(LO)] respectively does not provide additional information regarding the crystalline structure, as they are usually present in the Raman spectra of barium titanate-based materials, including at temperatures above the Curie temperature (in the paraelectric state), where normally all vibrational modes would be expected to collapse [42].

For the SG-CS ceramic, conventionally sintered from the starting sol-gel powder, the representative mode for the tetragonal phase at 294 cm^−1^ frequency is almost missing, suggesting that this specimen could be found in its paraelectric state. However, the presence of the small feature located at 181 cm^−1^ and assigned to the [*A*_1_(TO) + *E*(LO)] mode indicates that a certain orthorhombic polar order persists in this sample (Figure 5b). These data suggest that decreasing grain size may induce both the shift of the Curie temperature towards lower temperature values than the ambient temperature and a broader ferroelectric-paraelectric phase transition, accompanied by an overlap in a larger temperature range of some dissimilarly distorted phases induced by higher internal stresses acting in this submicron-structured ceramic compared to those specific to the coarser SS-CS sample. Further measurements of the temperature-dependent dielectric response are required in order to sustain this assumption.

For the sol-gel nanocrystalline ceramic consolidated by plasma spark sintering (SG-SPS), the drop in the intensity of the [*A*_1_(TO)] mode and the overall flattening of the Raman spectrum compared to those corresponding to microstructured sol-gel ceramics obtained by conventional sintering, indicate the prevalence of the cubic phase specific to the paraelectric state (Figure 5c). However, it is worth mentioning that the “silent” [*E*(TO) + *E*(LO) + *B*_1_] mode located at 294 cm^−1^ and, especially, the [*A*_1_(TO) + *E*(LO)] mode at 181 cm^−1^, are more visible than those corresponding to the submicron-structured SG-CS sample, which seems to suggest that the residual polar order above the Curie temperature is more stable as the grain size decreases toward the nanometer scale. This behavior was also reported for the fine-grained Ba_0.6_Sr_0.4_TiO_3_ ceramics, which at room temperature are found in the paraelectric state [36] and supports the hypothesis of an enhancement of a relaxor-like behavior determined by a particular structure of the grains, which consist of a non-polar, cubic matrix in which polar nanoregions (PNR) are randomly distributed. Complex distortions may arise in these polar nanoclusters, i.e., tetragonal, orthorhombic, or rhombohedral.

In the Raman spectra of the fine-grained SG-CS and SG-SPS ceramics, two additional, high-frequency modes of low intensity, located at 787 cm^−1^ and 828 cm^−1^, specific to the homovalent B-site (Zr^4+^ ↔ Ti^4+^) and A-site (Ca^2+^ ↔ Ba^2+^) substitutions in the perovskite lattice, were also identified (Figure 5b,c). The higher substitution degree on the A-site (30% CaTiO_3_ in BaTiO_3_) compared to that on the B-site (20% BaZrO_3_ in BaTiO_3_) may be responsible for the somewhat higher intensity of the mode located at 828 cm^−1^ compared to that at 787 cm^−1^.

**Figure 6:**
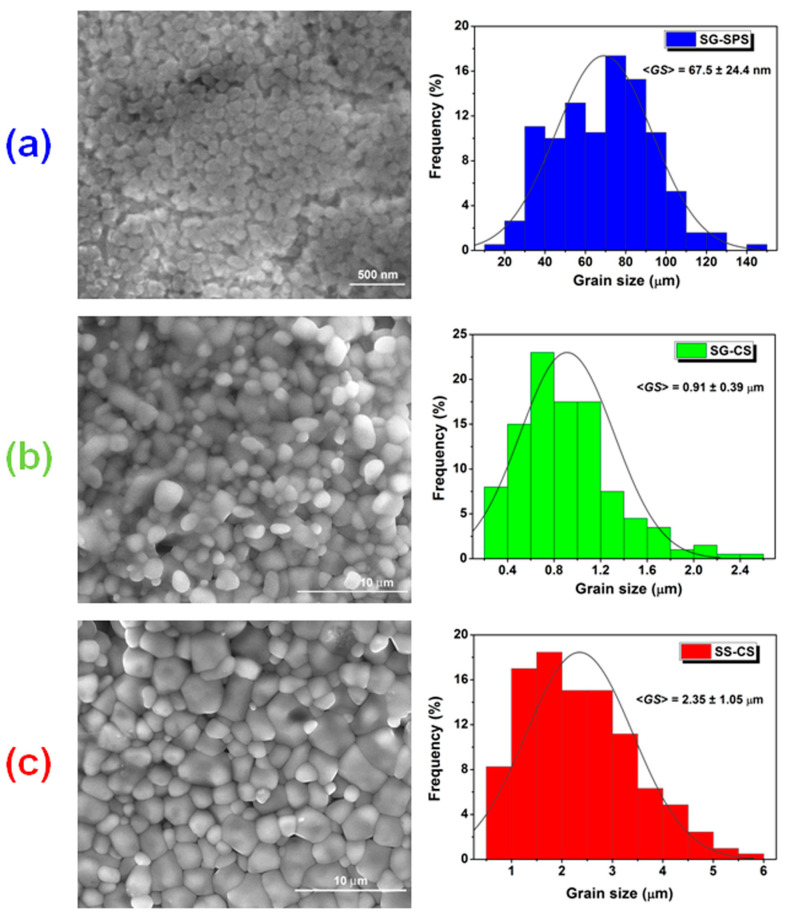
SEM images and the distribution of the grain sizes for BCTZ ceramics: (**a**) SG-SPS; (**b**) SG-CS; (**c**) SS-CS.

The FE-SEM images of Figure 6 reveal the microstructure on the polished cross-sections of the BCTZ ceramics after thermal etching. One can notice that highly densified ceramics with easily distinguishable equiaxial grains were obtained, irrespective of the synthesis method and/or consolidation technique.

Conventional sintering produced microstructured and submicron-structured BCTZ ceramics with average grain sizes <*GS*> of 2.35 μm for the SS-CS sample (Figure 6c) and 0.91 μm for the SG-CS ceramic (Figure 6b), while after spark plasma sintering a nanocrystalline specimen (SG-SPS), with <*GS*> = 67.5 nm was obtained (Figure 6a). The average grain size values of the ceramics under investigation are also listed in Table 1. The grain size value in the nanometer range obtained for the SG-SPS sample under investigation is quite different from that of 0.4 μm reported by Hao et al. [27] for a BCTZ ceramic of similar composition and also consolidated by SPS. This can be explained in terms of the different reagents (commercial oxides and carbonates in the first case and sol-gel nanopowder in our study) and dissimilar spark plasma sintering conditions (1120 °C/5 min—Hao et al. and 1050 °C/4 min—present work) used to produce these fine-grained BCTZ ceramics. All the ceramics under investigation exhibit unimodal grain size distributions, whose maxima become narrower (indicating an increasingly higher microstructural uniformity) when grains are downscaled from the micro toward nanometer range (see the grain size distribution histograms of Figure 6a–c).

The relative density values of the BCTZ samples under investigation decrease in the following order: SG-SPS > SS-CS > SG-CS, as indicated in Table 1. As expected, for the ceramic SG-CS sample, the relative density (ρ_r_ = 84.8%) is lower than that (ρ_r_ = 97.9%) of the SG-SPS specimen, taking into account that the spark plasma sintering process involves field-assisted simultaneous pressing and heating, thus resulting in a higher densification. On the other hand, the higher density (ρ_r_ = 97.1%) in the SS-CS sample relative to that of the SG-CS specimen could be explained in terms of a higher density of the related green pellet, determined by the higher ability towards pressing of the mixture of conventional reagents (oxides and carbonates), with respect to that specific to the sol-gel nanopowder. It was found that, for similar conventional sintering conditions (1400 °C/4 h), the coalescence of the ceramic grains and the migration of grain boundaries, both caused by the grain growth process, is enhanced in the SS-CS sample. The higher sinterability results in a lower intergranular porosity and, consequently, better densification in the SS-CS sample compared to the SG-CS ceramic.

### 3.2. Low-Field Dielectric Properties

**Figure 7:**
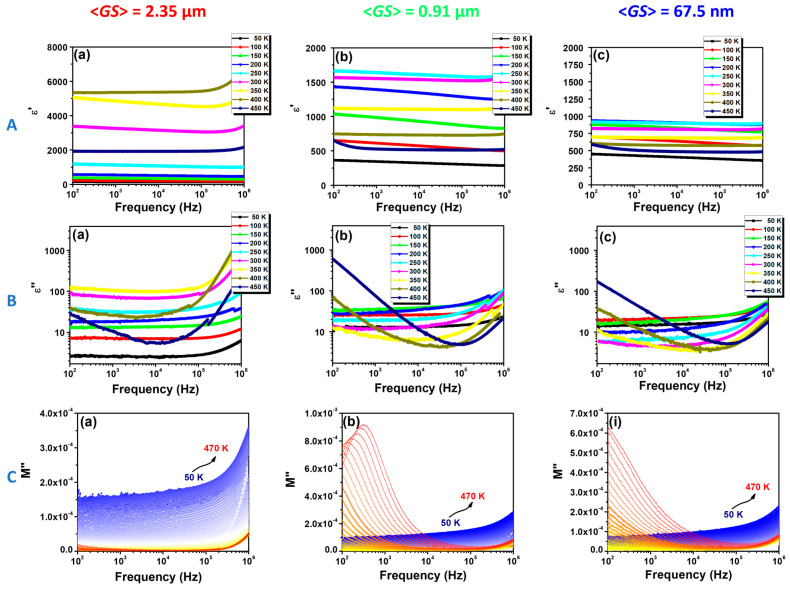
The frequency dependence of: (**A**) the real part of the permittivity *ε*′(ν), (**B**) the imaginary part of the permittivity *ε*″(ν), and (**C**) the imaginary part of the dielectric modulus *M*″(ν) for the BCT-BZT ceramics prepared and consolidated by alternative methods—(**a**): SS-CS (<*GS*> = 2.35 μm); (**b**): SG-CS (<*GS*> = 0.91 μm); (**c**): SG-SPS (<*GS*> = 67.5 nm).

The BCTZ ceramics exhibit distinct frequency dependence of the real ε′(ν) and imaginary ε″(ν) components of permittivity, as well as of the imaginary part of the dielectric modulus, M″(ν), which can be observed in the dielectric data shown in Figure 7.

The relationship between the frequency dependence of dielectric permittivity reveals the mutual influence of microstructural characteristics and dielectric response. This interrelation provides insights into the intrinsic behaviors of materials under alternating electric fields, enabling more precise manipulation of material properties. Ceramics of varying grain sizes display unique characteristics in the frequency dependence of their dielectric properties, showing distinct behaviors across different frequency ranges. Thus, SS-CS exhibits a relatively uniform dielectric permittivity within frequency domains below 10^5^ Hz and at moderate temperatures, which is an indicator of the stability of dipole-electric interactions in the material structure. However, at higher frequencies and under elevated temperature conditions, especially near the Curie Point, a significant amplification of the ε′(ν) is observed (Figure 7A(a)). This significant change in dielectric properties can be attributed to the concurrent phenomena of polarization and dielectric relaxation, where the alignment of the electric dipoles becomes inhibited by the dynamics of the system, resulting in an increased accumulation of energy within the material. The underlying processes of polarization may manifest through the orientation of dipoles in the direction of the applied electric field, a process which at high frequencies may become inefficient due to the reduced time available for reorientation, thus leading to an increase in the energy stored in the electric field and, consequently, the rise of the dielectric permittivity. The contrasting dielectric behaviors observed in the compositions with different microstructures SG-CS and SG-SPS can be meticulously attributed to the interplay of grain size and environmental conditions (Figure 7A(b,c)). Dielectric permittivity decreases from thousands down to hundreds, and different polarization phenomena occur. Conversely, fine-grained ceramics exhibit a marked increase in dielectric permittivity, especially at higher temperatures and lower frequencies. This behavior is indicative of the Maxwell–Wagner effect, where the abundance of grain boundaries and interfaces facilitates electric charge accumulation. This accumulation induces interfacial polarization and a corresponding enhancement in stored energy. Particularly under conditions where reduced frequency provides ample time for charge migration and accumulation at grain boundaries and increased temperature facilitates the mobility of ions and defects within the material, this fine-grained microstructure modifies the material’s response to applied electric fields. This interplay between microstructure and response not only provides avenues for advanced applications but also opens up opportunities for innovative technological solutions in the realm of electronic materials. By understanding and leveraging these nuanced behaviors, new pathways can be discovered for the optimization and application of electronic materials in varying technological contexts.

On the other hand, it can be seen that the loss factor ε″(ν) for SS-CS increases at high frequencies even at temperatures exceeding the stability range guaranteed by the Curie point in the phase transition region (Figure 7B(a)). This can be interpreted as a manifestation of increased dynamics of the material structure at the atomic and molecular level. In this enhanced thermal context, dislocations and structural fluctuations not only persist but also intensify, inducing a regime of high energy loss. This phenomenon is concretely reflected in the amplification of the values corresponding to the imaginary part of the permittivity, with substantial implications on the overall dielectric behavior of the material. At the same time, the SG-CS and SG-SPS specimens present a reduced loss factor ε″(ν) (Figure 7B(b,c)), where the Maxwell–Wagner effect predominates. This effect typically occurs in materials with heterogeneous structures, like those with differences in grain sizes or phases, leading to the development of interfacial polarization at the grain boundaries due to the accumulation of charge carriers, a phenomenon intensified by the presence of the substantial number of grain boundaries and interfaces in these specimens. In this context, the grain size plays a key role in defining the dielectric response of the material. Specifically, the increased number of grain boundaries in fine-grained specimens acts as a facilitator for charge accumulation and interfacial polarization, contributing significantly to the Maxwell–Wagner effect. This contrasts with larger-grained specimens, where fewer interfaces are available for charge accumulation, reducing the prominence of the Maxwell–Wagner effect and hence the loss factor ε″(ν).

The dielectric modulus is commonly used to explain bulk relaxation properties in doped BaTiO_3_-based ceramics. The complex dielectric modulus is defined as [43]: M*(*f*) = *M*’(*f*) + *iM*″(*f*), where: *M*’(*f*) = *ε*′(*f*)/(*ε*′^2^(*f*) + *ε*″^2^(*f*)), and *M*″(*f*) = *ε*″(*f*)/(*ε*′^2^(*f*) + *ε*″^2^(*f*)).

From the dispersion of the imaginary part of the dielectric modulus *M*″(*f*), useful information concerning the charge transport mechanisms, such as electrical transport and conductivity relaxations, can be obtained. A conductivity relaxation is described by the presence of a peak in *M*″(*f*) spectra and no peak in the corresponding plot of *ε*”(*f*), while the dielectric relaxation gives maxima in both the *ε*”(*f*) and *M*″(*f*) dependences. For the coarser-grained SS-CS BCTZ ceramic sample, with an increasing frequency above 10^5^ Hz, for all temperatures, but especially at low-temperature values, the beginning of a sharp increase in *M*”(ν) (Figure 7C(a)), very similar to that in ε″(ν) (Figure 7B(a)), can be noticed. This increase seems to suggest the upward branch of a dipolar relaxation. For the finer-grained SG-CS and SG-SPS samples, as the grain size decreases, the rise of *M*″ is much less steep (Figure 7C(b,c)), which suggests that either the dipolar relaxation is strongly affected, or the related relaxation maxima are shifted at higher frequency values, far above 1 MHz. On the other hand, downscaling grain size toward the submicron and nanometre range results in the appearance of the Maxell–Wagner phenomena associated with the significant contribution of the increasingly higher density of grain boundary regions, whose *M*″ maxima are shifted to lower frequency values, i.e., (10^2^–10^3^) Hz for the submicron-structured SG-SC sample (Figure 7C(b)) and below 10^2^ Hz for the nanostructured SG-SPS specimen (Figure 7C(c)).

**Figure 8:**
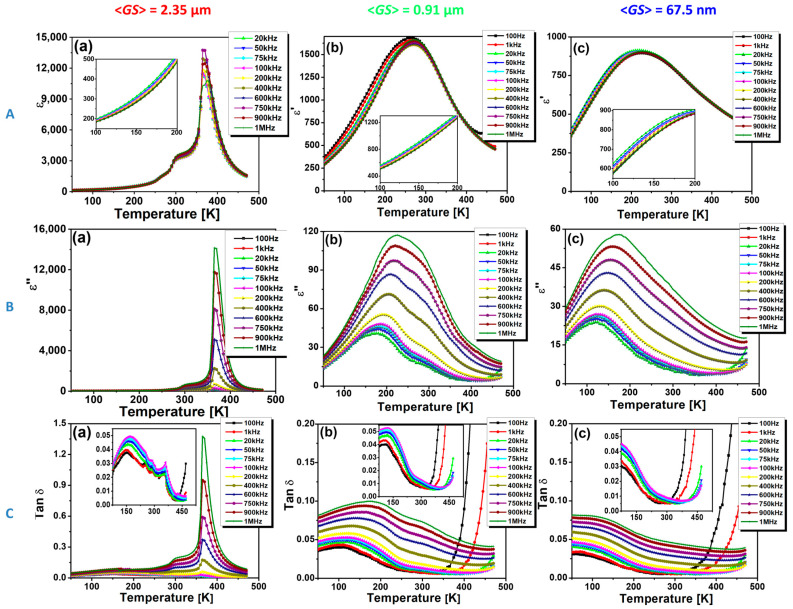
The temperature dependence of the (**A**) real part of the permittivity ε′(T), (**B**) the imaginary part of the permittivity, ε″(*T*), and (**C**) the loss tangent, tan δ(*T*) represented for a few selected frequencies for the BCT-BTZ ceramics prepared and consolidated by alternative methods—(**a**): SS-CS; (**b**): SG-CS; (**c**): SG-SPS.

The real part (ε′) and the imaginary part (ε″) of the permittivity, as well as the dielectric losses (expressed as tan δ) as a function of temperature, are presented in Figure 8, spanning a temperature range between 50 K and 470 K.

The BCTZ compositions SS-CS, SG-CS, and SG-SPS undergo phase transitions discernible in the plots of *ε′*(*T*), *ε*”(*T*), and tan δ(*T*) (Figure 8A–C). These transitions indicate altered structural and electronic configurations, leading to modified interactions and alignments of the atomic and molecular entities within the material. Thus, the coarse grain SS-CS specimen undergoes three phase transitions, which are observable in the temperature dependence of the real part of the permittivity, *ε′* (Figure 8A(a)). Thus, the strongly flattened feature on the *ε′*-*T* curve, barely detected at ~265 K, is assigned to the rhombohedral-orthorhombic (R-O) phase transition, while the more pronounced shoulder in the proximity of room temperature (300 K) is ascribed to the orthorhombic-tetragonal (O-T) phase transition, that displays a slight frequency-dispersive character. Zhou et al. account for the orthorhombic-tetragonal phase transition at low temperatures near RT for the observed dual nature of the electrocaloric effect (ECE) [44]. Subsequently, a further increase in temperature leads to a sharp peak around 365 K (92 °C), indicating the ferroelectric-to-paraelectric phase transition specific to the tetragonal-to-cubic change in the crystalline structure of the sample. The sharpness of this permittivity maximum at the Curie temperature (*T*_C_) is the signature of a first-order phase transition and indicates a significant realignment or restructuring at the atomic or molecular scale. After this temperature, a linear, steep drop in permittivity is observed, suggesting that the SS-CS sample is found in its paraelectric state. These findings are supported by previous studies on BCT-BZT ceramics, which have investigated the phase transitions and electrical properties of these materials [9]. The significant decrease in grain size toward the submicron and nanometer range, as observed in the SG-CS (Figure 8A(b)) and SG-SPS (Figure 8A(c)) specimens, results in a distinct change in the temperature-dependent permittivity behavior. Unlike the coarse-grained SS-CS specimen, the fine-grained ceramics SG-CS and SG-SPS ceramics exhibit only a single broad maximum of permittivity. Earlier, this was reported usually to occur in homovalently B-site substituted barium titanate solid solutions, where above a certain solute content, due to the opposite evolution vs. temperature of *T*_C,_ on the one hand, and of the temperatures corresponding to the lower-temperature phase transitions O-T (*T*_1_) and R-O (*T*_2_), on the other hand, the three permittivity peaks become closer and closer, until they coalesce into a single broad maximum, specific to the so-called “pinched” phase transition [45,46]. Such a phenomenon was also revealed in fine-grained undoped BaTiO_3_ ceramics, when the permittivity maximum becomes broader and increasingly flattened, and its temperature is gradually shifted towards lower temperature values as the grain size decreases toward the nanometer range [29,30,31,32,33]. This finding could be explained in terms of an increased surface-to-volume ratio, leading to more pronounced interfacial or boundary effects due to the inner stress and it is in good agreement with the results obtained for undoped BaTiO_3_ ceramics [29,30,31,32,33], as well as with the data reported by Orlik et al. [23] for their BCTZ ceramics with different grain sizes obtained by conventional sintering and microwave sintering in various conditions, respectively. On the other hand, unexpectedly, Hao et al. [27] found an increase in the Curie temperature with the grain size decrease, which was explained by taking into account the “unit cell volume effect” induced by structural distortions [47,48]. One can conclude that this apparent discrepancy in the variation of *T*_C_ with decreasing grain size originates in the competition between two factors acting with opposite effects, i.e., inner microstructural stress and unit cell volume shrinkage. The single, broadened maximum may also indicate a more homogenized response across the grain boundaries or a wider range of thermal activations, obscuring the distinct phase transitions [49].

The temperature values corresponding to the permittivity maxima, *T*_m_, are 270 K for SG-CS and 220 K for SG-SPS, respectively. Compared to the coarser SS-CS sample, the permittivity maxima dramatically dropped from 11,696 to 1626 for the SG-CS and 921 for the SG-SPS, respectively, at a frequency of 100 kHz. At room temperature, permittivity (ε′) values of 3056, 1522, and 804 and dielectric losses (tan δ) of 2.9, 1.3, and 0.9% were found for the BCTZ ceramics under investigation, i.e., SS-CS, SG-CS and SG-SPS, respectively. A slight frequency dispersion, increasingly pronounced with decreasing grain size in the low-temperature range, suggests an incipient ferroelectric-relaxor crossover (insets of Figure 8A(a–c)). These findings show that the decrease in grain size towards the nanometer range leads to the widening of the temperature range, i.e., (225–325) K in SG-CS and (150–350) K in SG-SPS ceramic, in which overlap of crystalline phases with different symmetries of the unit cells occurs, thus contributing to better thermal stability of the dielectric response. The results of the dielectric measurements sustain the assumptions regarding the structural characteristics formulated based on the Raman spectroscopy data recorded at room temperature.

With decreasing grain size in the BCTZ ceramics, the values of both the imaginary part of the dielectric constant and the dielectric losses expressed as ε″ and tan δ seem to decrease significantly (Figure 8B(a–c) and Figure 8B(a–c), respectively). Thus, at the highest frequency of 1 MHz, the dielectric losses of the SS-CS ceramic sample show a maximum at the Curie point of 140%, which drops below 10% for SG-CS and maintains values below 8% for SG-SPS in the entire temperature range (Figure 8B(a–c)). However, at lower frequency values (100 Hz–100 kHz), the temperature dependence of the dielectric losses is quite different. Thereby, the insets of Figure 8B(a–c) reveal that, over this frequency range, at temperatures below the ferroelectric-paraelectric phase transition (<400 K for SS-CS and <300 K for SG-CS and SG-SPS), the maximum values of the dielectric losses are below 5.5% and decrease in order SG-CS (5.3%) > SS-CS (5.0%) > SG-SPS (4.7%), when both the compactness (see the relative density values of Table 1) and microstructural uniformity of the ceramic samples increases. At higher temperatures and, especially for the lower frequencies, the dielectric losses strongly increase in the fine-grained SG-CS and SG-SPS ceramics due to the Maxwell-Wagner phenomena associated with an increasingly higher density of grain-boundary states (insets of Figure 8B(a–c)). For the coarser SS-CS sample, the abnormal increase in both the imaginary part of the dielectric constant (ε″) (Figure 8B(a)) and the dielectric losses (Figure 8C(a)) recorded in the temperature range around the ferroelectric-paraelectric phase transition at frequencies above 200 kHz could be associated with some dipolar relaxation phenomena. This observation is in good agreement with Figure 7B(a), which revealed for the conventionally prepared specimen a steep increase in ε″ around the Curie temperature as the frequency increases above 10^5^ Hz. This feature might be related to the upward branch of the dipolar relaxation, whose maximum could be found at frequency values above 1 MHz (the highest frequency used for the dielectric measurements in this study).

**Figure 9:**
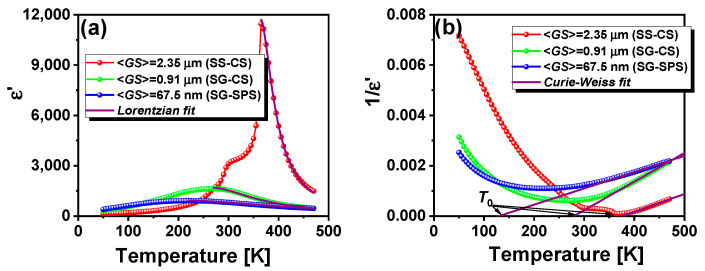
The temperature dependence of: (**a**) the real part of permittivity ε′(T) for different grain sizes represented for a fixed frequency of 100 kHz and (**b**) Curie–Weiss analysis.

For a clearer and more detailed visualization, the temperature dependence of permittivity for each sample at a fixed frequency of 100 kHz has been plotted. Figure 9a illustrates the variation in permittivity as a function of temperature for BCTZ ceramics of different grain sizes, allowing a direct comparison of the dielectric behavior between the three samples under investigation. The study is based on the Curie–Weiss law, an empirical equation describing the dielectric behavior of materials near the transition temperature, which can be used to extract important information about internal dynamics and interactions in the system. For typical ferroelectrics in the paraelectric phase above the *T*_C_, the dielectric permittivity obeys the Curie–Weiss law [50,51]:

(1) $$\frac{1}{\varepsilon }=\frac{T-T_{0}}{C},$$

where *ε* is the real part of dielectric permittivity, *C* is the Curie constant, *T* is the absolute temperature, and *T*_0_ is the Curie–Weiss temperature. Curie–Weiss analysis provides an overview of the paraelectric behavior of the composite. However, experimental observations also revealed a frequency dispersion that increases with a decrease in <*GS*>. To elucidate additional insights into the nature of the dispersion and the degree of diffusivity of the diffuse phase transition, the dielectric data are analyzed with the Lorentzian formula [52,53]:

(2) $$\frac{\varepsilon_{A}^{\prime }}{\varepsilon }=1+\frac{{\left(T-T_{A}\right)}^{\gamma }}{2\delta^{2}}$$

in which *ε*_A_ is the maximum permittivity at the corresponding temperature *T*_A_, *δ* describes the wide temperature range of the phase transition, and *γ* is the diffuseness degree (1 ≤ *γ* ≤ 2, *γ* = 1 for typical ferroelectrics and *γ* = 2 for “complete” relaxors). *ε*_A_ and *T*_A_ are determined by solving the equation and may have different values than the maximum permittivity *ε*′_m_ measured at its corresponding temperature. The Lorentzian and the classical Curie–Weiss analyses are shown in Figure 9a,b (purple lines represent the fits using Equations (1) and (2), respectively), and the results are listed in Table 2.

As the grain size decreases, not only *T*_C_/*T*_m_ but also *T*_0_ shifts progressively to lower values, from 377 K (SS-CS) to 280 K (SG-CS) and 130 K (SG-SPS). The shift in the Curie point can be attributed to factors such as interfacial strain, defect density, and internal stresses, which inherently vary as a function of microstructural features, especially of grain size, and highlight the significant influence of grain size on the ferroelectric properties of the material.

The Curie constant describes the behavior of ferroelectric materials near their *T*_C_, and the first assumption is that they should act linearly with decreasing granular size. Zhao et al. found the Curie–Weiss temperature in barium titanate ceramics to gradually decrease with decreasing grain size. The Curie constant exhibits almost similar values for the micron and submicron-structured samples and even lower for the nanostructured specimen with a grain size <*GS*> ~50 nm [29]. The value of the Curie constant decreases monotonically with increasing grain boundary thickness, although it is independent of the low-permittivity grain boundaries [54]. Conversely, Tanga et al. obtained BCZT ceramics with different grain sizes between 0.85 and 30 μm, an inversely proportional relationship between grain size and Curie constant [55]. A clear trend of decreasing the Curie constant with a decrease in density was obtained elsewhere [56]. A strong correlation between the Curie constant and the density of BCTZ samples was observed. In this case, the variation in the Curie constant is more strongly influenced by the sample density than the grain size. Higher density involves improved connectivity between the grains and a more efficient polarization, even if the grains are structured at the nanometer scale (SG-SPS). This could counterbalance effects associated with grain size, such as distortions in the grain boundary regions, leading to higher values of the Curie constant.

**Figure 10:**
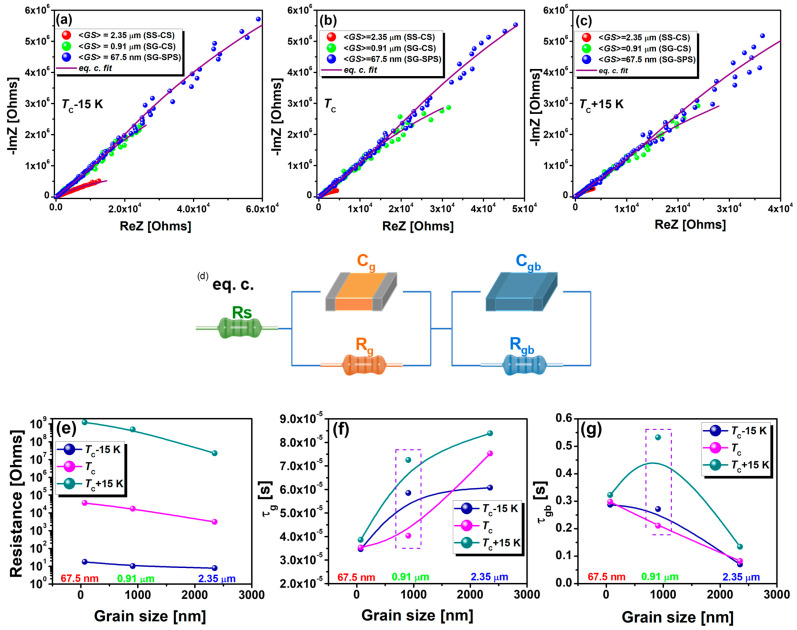
Nyquist plots of the complex impedance recorded (**a**) below the Curie point (*T*_C_ − 15 K), (**b**) at the Curie point (*T*_C_), and (**c**) above the Curie point (*T*_C_ + 15 K) for each of the BCT-BZT compositions (the solid red lines are the fitting results based on shown equivalent circuit in (**d**); (**d**) the equivalent circuit used to fit the experimental data, (**e**) the resistance parameters calculated for the grains and the grain boundaries, the time constant ascribed to (**g**) the grains and (**f**) the grain boundaries.

Impedance spectroscopy analysis has been employed to understand this tendency. Figure 10 shows the Nyquist plots based on the real (ReZ) and imaginary (-ImZ) parts of the impedance recorded at different temperature set points from 50 K up to 470 K in the 10^2^ Hz–10^6^ Hz frequency range. The interpretation of the Nyquist plot can be complex, as it might reflect contributions from multiple system components and phenomena, which include ceramic resistance, capacitance of the electrode/ceramic interface, and the diffusion of species within the system. In the Nyquist plots, a pronounced transformation in the semicircular arc is evident. Specifically, as the grain size diminishes, the semicircular arc ascribed to the grain boundaries at low frequencies increases and adopts a more depressed shape. Additionally, distinct changes in the arc’s shape are observed at different temperature points: below *T*_C_, at *T*_C_, and above *T*_C_ (Figure 10a–c). To glean insightful information about the processes within the system, we used the standard equivalent circuit (Figure 10d) typically applied to polycrystalline ceramics with metal electrodes. This equivalent circuit consists of a resistance (*R*_s_) associated with electrodes and external circuitry, connected in a series of two R-C branches ascribed to the grain volume and the grain boundaries. Each branch contains a single resistor (*R*_g_ for grains and *R*_gb_ for the grain boundaries) and capacitor (*C*_g_ for grains and *C*_gb_ for the grain boundaries) elements connected in parallel. This configuration is used to model and understand the complex impedance behavior arising in different regions of ceramics, namely grains and grain boundaries, each contributing differently to the overall impedance of the ceramic material. The resulting parameters are listed in Table 3.

The series resistance *R*_S_ arises due to parasitic resistance losses attributable to an increased roughness or reduced adhesion between ceramics and electrodes and acts as a shift in the Nyquist plot along the real axis away from the origin [57]. In BCTZ ceramics, *R*_S_ slowly increases with a decrease in grain size. In addition, a small decrease in the *R*_S_ value can be observed with increasing temperature, from *T*_C_ − 15 to *T*_C_ and *T*_C_ + 15 in the case of micron- and submicron-structured ceramics. The *R*_S_ values for the nanostructured sample tend to remain temperature invariant. The parameters derived for the elements in the equivalent circuit associated with grains and grain boundaries are examined further. The resistances *R*_g_ attributed to the core of the grains are consistently lower than those of the grain boundaries *R*_gb_ across all samples, reflecting high dielectric susceptibility and, therefore, high conductivity at the grain level. Therefore, regardless of grain size, BCTZ specimens exhibit highly resistive grain boundaries and grains with lower resistivity (Figure 10e). As the grain size decreases, both the grain and grain boundary resistances *R*_g_ and *R*_gb_ are increasing, primarily due to a higher density of grain boundaries and, consequently, to the enhanced influence of surface effects in smaller grains. At the same time, the associated capacitances *C*_g_ and *C*_gb_ calculated based on the equivalent circuit decrease with grain size decreasing, both for grains and grain boundaries, which is in agreement with what was experimentally determined. In the advanced analysis, the circuit branch corresponding to the grains manifested a rapid time constant, τ_g_ of ~10^−5^ s, whereas the branch attributed to the grain boundaries exhibited a markedly slower time constant, τ_gb_ of ~10^−1^ s, signifying a pronounced disparity in the temporal response mechanisms between the grains and their boundaries. As grain size increases, the reduced surface-to-volume ratio accentuates the influence of intrinsic bulk properties over surface effects. This, coupled with longer diffusion lengths in larger grains, inherently amplifies the time constants due to extended charge carrier dynamics. Thus, a slow increase in the time constant ascribed to the grains is visible in Figure 10f. On the other hand, as grain size increases, there is a reduction in grain boundary density, which means fewer interfaces for charge carriers to interact with over a given volume; concurrently, the larger grains entail longer inter-granular distances but fewer boundaries to traverse, resulting in swifter charge carrier transitions (paths) across these interfaces, thereby decreasing the grain boundary time constant (Figure 10g). However, a deviation can be observed in the case of the SG-CS specimen, for which a very high value of the time constant assigned to the grain boundaries was calculated, contrary to expectations and in contrast to the other samples (Figure 10g). Specifically, the significantly higher value of the time constant for the grain boundaries in the SG-CS specimen, when compared to other samples, is eminently attributable to the porosity that negatively affects the charges migration, thus leading to longer time constants at the grain boundaries at temperatures above and below *T_C_*. At *T_C_*, the time constant for grain boundaries decreases almost linearly with decreasing grain size, suggesting that the structural changes dominate over the variation in electrical conductivity between grain volume and grain boundaries.

## 4. Conclusions

Dense, lead-free 0.5Ba_0.7_Ca_0.3_TiO_3_–0.5BT_0.8_Zr_0.2_TiO_3_ (BCTZ) ceramics of various grain sizes, ranging from microns toward nanometres were successfully produced by using alternative synthesis methods (solid-state reaction and sol-gel) and consolidation techniques (conventional and spark plasma sintering, respectively). At room temperature, the crystalline structure of the conventionally sintered BCTZ ceramics is orthorhombic, while the SP-sintered specimen exhibits a mixture of orthorhombic, tetragonal, and cubic polymorphs, corresponding to the major perovskite phase. Decreasing grain size strongly affects the nature of ferroelectric-paraelectric phase transition, which gradually changes from a first-order type, characterized by a sharp, high-value permittivity maximum, ε_m_, toward “pinched,” diffuse phase transitions of second order and related broad permittivity maxima whose values steeply drop below 1000. It was found that the increasing diffuseness and the more pronounced flattening of the permittivity maximum and the shift of its temperature *T*_m_ toward lower temperature values are caused by both the internal stress associated with the microstructure refinement and the coexistence of dissimilarly distorted polymorphs over an increasing temperature range. Moreover, the slightly higher frequency dispersion at temperatures below *T*_m_, especially for the SP-sintered BCTZ specimen, suggests an incipient ferroelectric-relaxor crossover. At frequency values *f* ≤ 100 kHz, the grain size decrease also induces the decrease in the dielectric losses, tan δ, to values below 1% for the nanocrystalline BCTZ specimen. Permittivity values below 1000, low dielectric losses, and high thermal stability of the dielectric response are requirements imposed by using microwave devices, which are fulfilled by the nanostructured BCTZ ceramic sample. It was found that grain size does not affect the Curie constant, but it influences the evolution of the time constants corresponding to both the grain core and the grain boundary regions.

In summary, this research opens doors to innovative materials with tailored dielectric properties, enabling advancements in various technological applications where precise control over dielectric responses is a key factor. Further exploration in this field promises to unlock even more possibilities for designing materials that meet the specific requirements of emerging technologies.

## Figures and Tables

**Table 1:** Structural characteristics of the obtained ceramics.

Characteristics	Ceramic Sample								
	SS-CS	SG-CS			SG-SPS				
**Identified phase**	*BCTZ	BCTZ	Ba_2_TiO_4_	BaTi_2_O_5_	BCTZ			Ba_2_TiO_4_	BaTi_2_O_5_
**ICDD PDF4 +**	01-086-8337	01-086-8337	04-006-8930	04-017-1818	01-086-8337	00-072-0077	00-070-0583	04-006-8930	04-017-1818
**System/** **Space group**	Orthorhombic Amm2	Orthorhombic Amm2	Monoclinic P21/n	Orthorhombic Pnma	Orthorhombic Amm2	Tetragonal P4mm	Cubic Pm-3m	Monoclinic P21/n	Orthorhombic Pnma
**Phase composition (%)**	100	96.0	0.3	3.7	58	0.1	27.9	5.2	8.8
***a* (Å)**	3.995136 ± 0.000258	4.014679 ± 0.000148	6.077217 ± 0.000580	10.288960 ± 0.000555	4.026096 ± 0.000386	3.582892 ± 0.001127	4.032959 ± 0.000332	6.105985 ± 0.000269	10.428280 ± 0.031393
***b* (Å)**	5.671437 ± 0.001329	5.672983 ± 0.000614	7.680506 ± 0.000622	3.899641 ± 0.000297	5.681889 ± 0.003457	3.582892 ± 0.001127	4.032959 ± 0.000332	7.685928 ± 0.000298	3.954501 ± 0.008606
***c* (Å)**	5.668867 ± 0.001324	5.675007 ± 0.000475	11.238200 ± 0.001730	10.927010 ± 0.000565	5.684521 ± 0.003102	4.206708 ± 0.002251	4.032959 ± 0.000332	11.913690 ± 0.000562	10.717930 ± 0.033939
***V* (Å^3^)**	128.4461	129.2495	469.0049	438.4272	130.0381	54.0019	65.5951	559.1114	441.9929
**Relative density,** ***ρ*_r_ (%)**	97.1%	84.79%			97.91%				
***R*exp**	7.62884	7.63566			8.4752				
***R*p**	8.30252	6.14165			6.63801				
***R*wp**	12.90004	9.11354			8.80129				
**χ^2^**	2.85934	1.42456			1.07843				
**Grain size, <*GS*> (μm)**	2.35 ± 1.05	0.91 ± 0.39			0.0675 ± 0.024				

*BCTZ—Ba_0.85_Ca_0.15_O_3_Ti_0.9_Zr_0.1._

**Table 2:** Experimental and fit parameters for BCT-BTZ ceramics.

Sample	<*GS*> nm	*γ*	*δ* (K)	*ε*′_A_	*ε*′_m_	*T*_0_ (K)	C	Slope	Intercept	*T*_C_ (K)
SS-CS	2350	1.67	13	11,696	11,457	377	1.4 × 10^5^	7 × 10^−6^	−2.67 × 10^−3^	365
SG-CS	910	1.71	40	1685	1626	280	8.7 × 10^4^	1.1 × 10^−5^	−3.19 × 10^−3^	270
SG-SPS	67.5	1.75	86	918	911	130	1.5 × 10^5^	6.5 × 10^−5^	−8.44 × 10^−4^	220

**Table 3:** The parameters obtained by employing the proposed equivalent circuit in the proximity of the Curie point.

Temperature	Sample	<*GS*> (nm)	*R*_S_ (Ω)	*R*_g_ (Ω)	*C*_g_ (F)	*R*_gb_ (Ω)	*C*_gb_ (F)	*τ*_g_ (s)	*τ*_gb_ (s)
***T*_C_ − 15**	SS-CS	2350	7.8	3134	1.9 × 10^−8^	2.3 × 10^7^	3.08 × 10^−9^	6.1 × 10^−5^	0.07
	SG-CS	910	10.3	17,457	3.4 × 10^−9^	4.9 × 10^8^	5.56 × 10^−10^	5.8 × 10^−5^	0.27
	SG-SPS	67.5	18	36,585	3.5 × 10^−10^	1.2 × 10^9^	2.32 × 10^−10^	3.5 × 10^−5^	0.29
***T*_C_**	SS-CS	2350	6.1	567	1.3 × 10^−7^	1 × 10^7^	8.1 × 10^−9^	7.5 × 10^−5^	0.08
	SG-CS	910	9.9	9915	4.1 × 10^−9^	3.8 × 10^8^	5.57 × 10^−10^	4 × 10^−5^	0.21
	SG-SPS	67.5	18.5	28,819	1.2 × 10^−9^	1.3 × 10^9^	2.32 × 10^−10^	3.5 × 10^−5^	0.3
***T*_C_ + 15**	SS-CS	2350	5.6	425	2 × 10^−7^	2.2 × 10^7^	6.14 × 10^−8^	8.4 × 10^−5^	0.13
	SG-CS	910	9.2	17,005	4.3 × 10^−9^	9.7 × 10^8^	5.48 × 10^−10^	7.3 × 10^−5^	0.53
	SG-SPS	67.5	18.9	25,270	1.5 × 10^−9^	1.4 × 10^9^	2.29 × 10^−10^	3.9 × 10^−5^	0.32

## Data Availability

Data are contained within the article.
